# Exploring the reporting standards of RCTs involving invasive procedures for assisted vaginal birth: A systematic review

**DOI:** 10.1016/j.ejogrb.2021.05.026

**Published:** 2021-07

**Authors:** Emily J. Hotton, Sophie Renwick, Erik Lenguerrand, Julia Wade, Tim J. Draycott, Joanna F. Crofts, Natalie S. Blencowe

**Affiliations:** a Translational Health Sciences, University of Bristol, Bristol, UK; b Southmead Hospital, North Bristol NHS Trust, Bristol, UK; c Population Health Sciences, University of Bristol, Bristol, UK; d Centre for Surgical Research, Population Health Sciences, University of Bristol, Bristol, UK; e University Hospitals Bristol NHS Foundation Trust, Bristol, UK

**Keywords:** Reporting standards, Complex interventions, Assisted vaginal birth, Ventouse, Forceps, Odon Device

## Abstract

**Objective:**

Assisted vaginal birth (AVB) is a complex intervention involving medical devices, comprising multiple components. This complexity creates difficulties when designing and conducting randomised controlled trials (RCTs), in terms of describing, standardising and monitoring the intervention, and accounting for differing clinician expertise. This review examines the reporting standards of complex interventions involving a medical device, in the context of AVB RCTs.

**Study design:**

Searches were undertaken from the start of indexing to March 2021, and limited to RCTs, feasibility and pilot studies including at least one device for AVB. RCTs were selected if they included participants having an AVB with any device, with or without a comparator group. Reporting details were assessed according to the Consolidating Standards of Reporting Trials extension for non-pharmacological treatments (CONSORT-NPT), focusing on intervention descriptions, standardization, adherence and clinician expertise. Screening of abstracts, full-text articles and data extraction was performed by two independent reviewers.

**Results:**

Of 4098 abstracts and 83 full-text articles, 39 papers were included, investigating 80 interventions. Twenty-seven different named devices were identified. Intervention descriptions were provided in 20 (55%) papers with varying levels of detail and with only one covering the entire procedure. Standardization of interventions was mentioned in 25 papers (64%). Only eight (21%) papers reported any form of adherence to the intended procedure. Some data regarding expertise were reported in 25 (64%) papers.

**Conclusions:**

Despite some compliance with reporting standards, there is a lack of detail regarding intervention description, standardization, adherence and expertise in RCTs of AVB. This creates difficulties in understanding how intervention delivery was intended and what actually occurred. Clearer guidelines for the reporting of invasive procedures and devices are required.

## Highlights

• Reporting standards of device RCTs are vital, ensuring correct implementation.
• Assisted vaginal birth interventions are complex and use devices to achieve birth.
• Reporting standards of assisted vaginal birth trials lack detail or clarity.
• Clearer reporting standards guidelines must be produced, ensuring transparent findings.

## Introduction

Assisted vaginal birth (AVB) is an invasive procedure that, in skilled hands, can markedly improve maternal and neonatal outcomes arising from complications in the second stage of labour [1]. Typically, AVB involves the use of devices such as forceps or ventouse and, more recently, new devices [2]. Well-designed randomised controlled trials (RCTs) are essential for assessing the effectiveness of invasive procedures such as AVB, thereby enabling clinicians to make evidence-based decisions about whether to introduce them in routine practice. Similarly, high-quality reporting of RCTs is necessary to ensure that such procedures and devices are adopted and implemented correctly. However, the design and conduct of RCTs involving invasive procedures and devices (such as AVB) can be challenging because of their complexity. Complex interventions are defined as those with multiple interacting components that can act independently or interdependently to influence outcomes [3]. This complexity can create difficulties in establishing how interventions should be delivered (standardization) and ascertaining whether they are actually delivered as intended (adherence) within an RCT. Additional challenges are that the delivery of complex interventions can be influenced by clinicians’ skill (expertise). There may also be repeated modifications of new invasive procedures or devices during the developmental phase, which may not be reported or accounted for in trial design, that may influence trial outomes [4,5].

With accumulating numbers of reports citing complications associated with new invasive procedures and devices, [[6], [7], [8]] expert panels have suggested that more rigorous clinical evaluation is required through improved RCT design [[9], [10], [11], [12]]. The need for methodological rigour has also been acknowledged in reporting guidance documents such as the Consolidating Standards of Reporting Trials extension for non-pharmacological treatments (CONSORT-NPT), which includes invasive procedures and devices such as those used for AVB [13]. CONSORT-NPT suggests that ‘precise details of the experimental treatment’, ‘details on whether and how the interventions were standardised’, ‘details of whether and how adherence of care providers to the protocol was assessed’, and ‘information about the expertise of care providers’ should all be described in trial reports [13]. As well as reducing bias and improving methodological quality, provision of this information should help to contextualise trial findings, enable successful interventions to be replicated in practice, and reduce research waste.

The aim of this review was to assess the reporting standards in RCTs of AVB according to CONSORT-NPT guidance, specifically focusing on intervention description, standardization, adherence and clinician expertise.

## Methods

Full methods have been published previously [14] and are summarised below. Patient and public involvement was not sought for this systematic review.

### Search strategy

Searches of Medline, EMBASE, The Cochrane Library (Cochrane Database of Systematic Reviews, Cochrane Central Register of Controlled Trials (CENTRAL), Cochrane Methodology Register, Cumulative Index of Nursing and Allied Health Literature (CINAHL) and ClinicalTrials.gov were undertaken from the start of indexing to 30th March 2021. Searches were limited to RCTs, feasibility and pilot studies including at least one intervention or device used for AVB (Table S1).

### Inclusion of papers

RCTs, pilot and feasibility studies (with or without a comparator group) were included. Retrospective studies (e.g. case-control or cohort studies) were excluded because CONSORT-NPT guidance focuses on reporting standards in RCTs. Simulation and animal studies were included if they otherwise met the inclusion criteria. There were no language restrictions. AVB devices were not limited to a single type or manufacturer. Any type of comparator was included (i.e. spontaneous vaginal birth (SVB), Cesarean section or another AVB device).

After removal of duplicate records, titles and abstracts of citations were screened for eligibility by two researchers (EJH and SR) using predetermined selection criteria. Reference lists of retrieved articles were manually searched for additional references, including published protocols for included studies. Each abstract was independently read by at least two authors and full-text articles meeting the inclusion criteria analysed by at least two authors (EJH, SR or NSB). Discrepancies were resolved by discussion with NSB.

### Data extraction

Data were extracted in the following categories: general study information, intervention description, standardization, adherence, and clinician expertise. The methodological quality of all included studies was assessed. Where available, information from citations to protocols or supporting material (such as pictures, videos or intervention manuals) was used to supplement data extraction. Data were extracted onto a customised data extraction form, independently, by at least two assessors for each paper (EJH and SR).

#### General study information

General study details including year of publication, study country of origin, study design, the number of participating centres and the total number of participants, were recorded.

#### Intervention description (CONSORT-NPT items 5 and 5a)

Reporting of details about the interventions was assessed by recording verbatim descriptions of the operative components and steps of the procedure (item 5a). A description was deemed to have been provided if anything more than the name of the intervention or device was reported. For example, a study reporting that ‘women had their birth assisted using forceps’ would not constitute a description, whereas information about how to apply the device to the fetal head would be considered as a description of the intervention. Although CONSORT-NPT requires ‘precise details’ of the intervention to be provided, this is not defined (item 5). Items 5 and 5a further stipulate the reporting of the *‘sufficient details to allow replication’* and of *‘different components of the intervention’.* We therefore defined ‘precise details’ of an intervention as provision of a description for each component of an AVB: device application, creation of a vacuum (ventouse only), device traction and device removal. Descriptions of co-interventions (defined as ‘naturally accompanying or associated elements to the intervention itself’ [15]) such as provision of analgesia was also recorded. Reporting of whether an intervention was described as novel was also recorded to understand whether ‘new’ interventions had better reporting details.

#### Intervention standardization (CONSORT-NPT item 5b)

Standardization was defined as the process of making an intervention conform to a standard (i.e. falling into an accepted range of quality). Reporting of standardization (of both interventions and co-interventions) was assessed by recording i) specific details about the criteria for using the device and ii) whether there was any permitted flexibility in this standardization, in terms of whether the operative components or steps were described as mandatory, prohibited, optional or flexible [16].

#### Intervention adherence (CONSORT-NPT item 5d)

Adherence was defined as the degree to which an intervention was conducted according to the protocol or outlined by its designers [[17], [18], [19], [20], [21]]. Any reporting of adherence to intervention or co-interventions was recorded, including details of how this was measured. Details about crossover from one intervention group to another were also recorded.

#### Clinician expertise (CONSORT-NPT item 15)

Provision of information about clinician qualifications, grade, number of AVBs previously undertaken (including those using the device under evaluation) and total number of births annually in the unit, were recorded. The number of clinicians undertaking AVBs in each trial group was documented, including whether they performed AVBs within one or more trial group. Information about study entry criteria for clinicians was extracted (e.g. supervision, eligibility or completion of pre-study training materials). Any descriptions or references to clinician learning curves were extracted verbatim.

#### Study quality

The Cochrane Risk of Bias tool was used to evaluate bias in all included RCTs [22]. Non-randomised pilot and feasibility studies were assessed by evaluating bias related to the process of trial recruitment, documentation of protocol non-adherence, reporting of a primary outcome, description of clear objectives and description of clear progression criteria [23].

### Data synthesis

Data were entered into a custom database. Any discrepancies in data extraction were resolved by discussion between EJH, SR and NB. The findings were tabulated and, where appropriate, descriptive statistics were performed. A narrative synthesis summarised the findings by organising the data into the relevant extraction themes. This review does not aim to make conclusions about the effectiveness of devices for AVB because the focus is on how clinical trials adhere to reporting standards for interventional research. Therefore, no meta-analyses were performed.

## Results

**Fig. 1:**
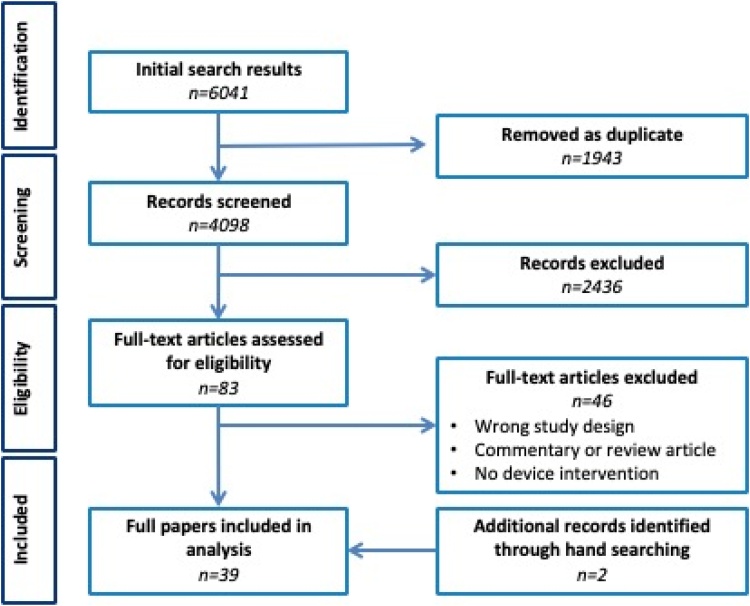
Flow diagram of papers throughout the systematic review, according to PRISMA criteria [43].

Titles and abstracts of 4098 articles were identified, 83 full text papers obtained and 39 eligible papers finally included. Two additional papers were identified from hand searching reference lists of included studies providing a total of 39 papers (Fig. 1).

### Study design and details

The 39 articles included 37 RCTs, one pilot study and one feasibility study, published between 1964 and 2020, across 22 different countries and included 8548 births from 8548 women and 8548 babies. Thirty-two (82%) were single-centre studies. Details about the type of AVBs evaluated in the studies is provided. There were a total of 80 interventions and comparators (39 device intervention groups, 37 device comparator groups and four SVB comparator groups). Nearly half of the papers (n = 19, 49%) compared different types or techniques of ventouse devices. There were no studies with a Cesarean section comparator group.

Thirty papers (77%) had two arms, each investigating a different device. A further two papers had three arms (ventouse-ventouse-forceps and ventouse-forceps-SVB) with only one paper having four arms (forceps in each). Four papers compared a device to SVB and the final two papers were single arm studies investigating a novel device for AVB, the Odon Device.

Two of the RCTs (5%) referenced earlier preliminary work and two further papers (5%) referenced study protocols. One of these provided further detail about adherence, but none provided additional information about intervention descriptions, standardization or expertise. The only feasibility study referenced earlier preliminary research and had a published protocol [2].

### Intervention description (CONSORT-NPT items 5 and 5b)

Of the 80 device intervention and comparator groups across the 39 studies, specific device name(s) were provided in 68 (35 intervention, 33 comparator), within which there were different brands of forceps (n = 10), ventouse (n = 16), and the Odon Device. The remaining eight stated only that ‘forceps’ (n = 4) or ‘ventouse’ (n = 4) were used.

Three papers provided a single sentence related to device use but these did not constitute intervention descriptions: *‘hospital guidelines for instrumental delivery were followed for all patients…’.* [24], ‘*for outlet forceps deliveries, we used classic instruments…and standard techniques and classification…’.* [25]*, ‘…standard* labor *room protocol was adhered to.’* [26]

Twenty papers (51%) provided some form of description of how to deliver at least one of the interventions; all but three relating to the ventouse (Table 1).

**Table 1:** Descriptions and standardisation of the co-interventions.

Component	Steps within component	Intervention description
Device application	Cup insertion	‘The rubber cup was introduced by squeezing it together and advanced towards the fetal head…’ [30]
	Check position	‘…and placed near the occipital fontanelle to ensure flexion of the vertex.’ [30]
	Tissue check	‘The maternal soft tissue was interposed between the cup and fetal scalp was cleared by digital examination.’ [30] [similar text for [44]]
Creating a vacuum	Support cup	‘With the cup gently pressed against the fetal head, the suction pressure…’ [30]
	Increase in pressure to 0.2	‘…vacuum was rapidly induced over 2−3 minutes. At the pressure of 0.2 kg/cm^2^ the application was checked…’ [45]
		‘An initial vacuum of 20 mmHg was applied for 1 min…’ [46]
	Tissue check	‘…vacuum was rapidly induced over 2−3 minutes. At the pressure of 0.2 kg/cm^2^ the application was checked…’ [45] and similar text for [46]
	Increase in pressure to 0.8	‘For both arms of the trial, the vacuum employed was 0.8 kg/cm^2^’ [35] and similar text for [30]
		‘The cups were attached to a hand-pump and the pressure was taken directly to 0.6−0.8 kg/cm^2^.’ [47] and similar text for [48]
		‘Negative pressure applied with the metal cup was 0.8 kg/cm^2,^ being gradually applied during 6 min, whereas with the Silastic cup, the pressure was immediately lowered to 1 kg/cm^2^’ [49]
		‘Malmström cup, vacuum was created gradually by increasing the suction by 0.2 kg/cm^2^ every 2 min until negative pressure of 0.8 kg/cm^2^ was reached. With the soft cup, negative pressure was increased to 0.8 kg/cm^2^ in 1 min’ [44]
		‘The vacuum pressure was then taken up directly to 45−50 mmHg.’ [46]
		‘The conventional stepwise method consisted of four incremental steps of 0.2 kg/cm^2^ every 2 min to obtain a final negative pressure of 0.8 kg/cm^2^. In the rapid methods the negative pressure of 0.8 kg/cm^2^ was applied in one step.’ [50] and similar text for [37]
		‘Pressure was maintained to 60 mmHg during traction in the uterus contraction.’ [51]
		‘The highest possible level of negative pressure (usually −0.8 kg/cm^2^) was obtained in a single step, usually requiring 1−2 minutes, with an electric vacuum pump controlled by a foot pedal.’ [52]
		‘For vacuum-assisted deliveries, pressure gradients of −54 to −58 cmHg were developed over 5−10 seconds…pressure was decreased to −10cmHg between contractions.’ [53]
		‘…the Malmstrom cup was applied, a vacuum was created gradually by increasing the suction by 0.2 kg/cm every 2 min until a negative pressure of 0.8 kg/cm was reached. With the soft Silc-cup, negative pressure was increased to 0.8 kg/cm in 1 min’ [54]
Device traction	Tissue check	‘The operator immediately made a check to ensure that maternal tissue was not included under the edge of the cup’ [55] similar text for [52]
	Monitor what?	‘The non-pulling fingers were applied on the foetal scalp to detect any leakage of seal. [46]
	Gentle traction with contraction	‘The first pull was with the next contraction.’ [47] similar text for [55]
		‘A continuous and even traction (in synchrony with uterine contraction) using the accoucher’s body weight, was applied with the pully elbow fully extended.’ [46]
		‘…the first pull was with the next contraction.’ [54]

Descriptions of forceps or ventouse assisted births were heterogeneous, with no papers providing ‘precise details’. The only paper that provided precise details was the feasibility study of the novel Odon Device [27]. In terms of the components for forceps or ventouse assisted births, some description was provided for device application (n = 2), creating a vacuum (n = 18), device traction (n = 11) and no information was provided about device removal. No papers provided intervention descriptions for forceps devices. The pilot paper reporting on the Odon Device provided both narrative and pictorial descriptions of how to use it [28] However, not all components were included and therefore the description was not considered as precise. However, the more recent feasibility paper on the Odon Device published the full instructions of use for the device, detailing every operative step with accompanying images [27].

The Odon Device papers were one of only four that explicitly stated that the device or device technique was new. Of the other two, one referenced previous work on the new device [29] and another provided detailed description of intervention delivery, discussing the majority (but not all) of the components [30].

Co-interventions were described in seven papers (18%) and consisted of: bladder care prior to device use (n = 5), maternal position (n = 3) and use of analgesia (n = 4) (Table 2).

**Table 2:** Details of co-interventions.

Co-intervention	Verbatim intervention description details
Bladder care	‘The bladder was emptied before application of the vacuum cup…’ [44]
	‘…the bladder was catheterised.’ [46]
	‘…the bladder was emptied before application of the cup.’ [30]
	‘…bladder empty.’ [33]
	‘…empty bladder.’ [27]
Maternal position	‘…placed in the dorsal lithotomy position with left lateral tilt.’ [29]
	‘The patient was put in the lithotomy position…’ [46]
	‘Position woman in lithotomy position.’ [27]
Analgesia	‘Continuous epidural anaesthesia…was provided to all patients after they entered the active phase of labour …’ [56]
	‘All patients received left lateral perineal infiltration with 20 ml 1% lidocaine.’ [44]
	‘The perineum was infiltrated with lignocaine 1%…’ [46]
	“Provide adequate analgesia according to facility procedures.’ [27]

### Interventions standardization (CONSORT-NPT item 5b)

Conditions for device use were reported in 38 papers (97%) although in 21 (54%) it was not clear whether these were the same across all study arms.

Information about any mandatory, flexible and optional operative components or steps was provided in 25 papers (64%). All except three related to ventouse, even when forceps were the intervention rather than the comparator (Table 3) [25,29,31].

**Table 3:** Details of device use standardisation for components of ventouse interventions: flexible (F), mandatory (M) and optional (O).

Component	Verbatim description of standardisation	
Choice of device (F)	‘…the exact type of cup to be used (i.e. whether silastic, metal, etc.), was at the discretion of the obstetrician performing the delivery…’ [35]	
	‘…range of metal cups was used (40−60 mm), Malmström, anterior, posterior Bird & ‘New Generation’ cups) depending on availability & operator preferences.’ [57]	
	‘The choice of which conventional cup [comparator] to use was then made by each individual clinician according to clinical judgement for each case.’ [24]	
	‘Three sizes of metal cups were used, 40, 50 and 60 mm in diameter. The rubber cups…were 50 and 60 mm in diameter.’ [58]	
	‘Decision for forceps delivery or ventouse extraction was of consultant and the choice of method was dependent entirely on his/her judgement.’ [31]	
Creating vacuum (M)	‘Negative pressure gradients were those suggested by the manufacturers…−0.2 kg/cm^2^ between contractions and -0.7 kg/cm^2^ for pulls…during contractions’ [59]	
Descent should be achieved with each contraction (M)	‘abandoned if station was not gained with traction or if any scalp trauma was noted…’ [29]	
	‘Descent should be seen with each traction…’ [24] and similar text [48]	
Delivery should be achieved with 3−4 contractions (M)	‘…without regard to the number of extraction attempts (“pulls”)…’ [29]	‘…traction efforts to deliver the baby were kept to less than three…’ [33]
	‘…three ‘pulls’ (one ‘pull’ being traction during a single maternal contraction)…)’ [57] and similar text [59]	‘According to departmental policy, a “freshman” is asked to stop the procedure after 3–4 pulls have failed to complete the ventouse delivery…’ [52]
	‘…4 pulls.’ [46]	
Cup displacement (M)	‘…vacuum extractor was considered to have failed when the cup fell more than twice…’ [51] and similar text [57]	
	‘Three cup disengagements (“pop offs”) mandated abandonment of the vacuum procedure.’ [29]	
	‘…one loss of suction…’ [59]	
	‘…the procedure should be abandoned if the cup detaches twice.’ [24]	
Cup reapplication (M)	‘one reapplication of the cup was permitted with further detachment indicating…a change in mode of delivery.’ [60]	
	‘Only two reapplications of the cup were allowed.’ [29]	‘If a cup slipped off, it was replaced once…’ [44]
	‘..only three reapplications of the cup were allowed.’ [33]	
Delivery should be achieved within 15 min of cup application (M)	‘The time limit of application was 15 min’ [47] and similar text [55]	‘restriction on the…duration of the extraction.’[52]
	‘…no longer than 20 min…’ [29]	‘…30 min after the beginning of traction attempts.’ [44]
Instrument failure (O)	‘If delivery with the selected instrument was not successful, the subsequent course of action (i.e. delivery with another instrument or caesarean section) was at the discretion of the obstetrician conducting the delivery.’ [35] and similar text [61]	

Seventeen papers (44%) reported flexibility in the devices clinicians were permitted to use, relating to the comparator group (n = 12), intervention (n = 1) and both intervention and comparator (n = 4). Five of the papers described flexibility in the specific brand of device that could be used, (ventouse = 4, forceps = 1), stating that the choice was left to the clinician. In a further study, women were randomised by clinician preference, ‘*Decision for forceps delivery or ventouse extraction was of consultant and the choice of method was dependent entirely on his/her judgement’.* [31]

Standardization was also reported for co-interventions in 12 papers (31%) and related to the provision of analgesia (*n* = 5) and use of episiotomy (*n* = 9).

### Intervention adherence (CONSORT-NPT item 5d)

Eight papers (21%) reported any form of adherence to the intended procedure. Of these, six stated that procedural details were documented by the clinician after the assisted birth; however, it was unclear whether this information related to details of how the procedure was undertaken or to maternal and neonatal outcomes following birth. One paper provided further information: *‘participants in the first phase were asked for written consent to [video] tape the application of the Odón device. These tapes were made available to the DSMB for safety evaluation purposes.’* However, no clarification was provided about who analysed the videos and the standards to which they were assessed [32]. The final paper of the Odon Device provided details of adherence in its published protocol; *‘an integrated qualitative study…will investigate: the practitioners’ use of the device to ensure that an appropriate training package is developed for trial; enable to intervention to be described and refined to optimise its use….’* [2]

Of the 37 papers with more than one trial group, 26 (71%) stated the number of women crossing over to another group. In a further eight (22%), it was unclear whether crossovers had occurred and in the remaining three (8%) there was no mention of crossovers.

### Clinician expertise (CONSORT-NPT item 15)

Some data were provided about the expertise of clinicians performing AVB in 25 papers (64%). None of the papers explicitly outlined the clinicians’ qualifications although their grade was reported in 23 papers (59%). One paper stated that ‘*trained registrars or specialist obstetricians performed all deliveries’* [33] but there was no clarification of the nature of this training. A further paper reported the number of AVBs previously undertaken by each clinician and grouped them according to whether they had performed fewer or greater than 20 procedures [34] however, it was not clear if they used the intervention or comparator to determine the grouping. Only eight papers (21%) reported the number of AVB performed per year in the trial centres. Although 15 (41%) reported the number of clinicians performing AVB in the study, none explicitly stated that the same clinicians performed interventions across all trial arms (where applicable).

Descriptions of the supervision of clinicians was provided in eight papers (21%); in the majority (*n=*7) this referred to trainees being supported by senior clinicians. The one pilot study stated that *‘all applications of the device were supervised, and assisted as required, by another obstetrician trained in the use of the device’* but did not specify what this training entailed [28]. Clinician eligibility criteria was mentioned in two papers (5%) *‘obstetric registrars (year 2 to year 4) trained in the use of the vacuum extractor’* [26] *and ‘each operator was classified according to prior experience with each instrument [vacuum extractor and forceps]’* [34]. None of the papers required clinicians to have completed a certain number of AVBs prior to joining the study. Only one paper provided detailed supplementary information regarding the training programme for operators [27]. Some information about pre-study training was reported in three papers (8%): *‘opportunity to watch a training video’* [35], *‘…verbal and written advice’* [26] *and ‘…instructed in the use of both methods…’* [34]. An additional paper clarified that *‘clinicians had no specific training in the use of the disposable cup before the trial’* despite the authors acknowledging that this was a new device [36].

Nine papers (23%) made reference to clinicians’ learning curve, three of which were in relation to devices that were described as new [[27], [28], [29]]. Of these nine studies, two accounted for the learning curve by comparing event rates across two time periods [35,36]. The remaining seven papers acknowledged that the learning curve may impact on study findings but did not adjust for this.

### Device success, failure and safety

Thirty-one papers (79%) reported device failure in either the intervention or comparator arms. Thirteen of these gave no details regarding the reported failures. The majority of the remaining papers gave very vague details regarding device failure. The feasibility study of the Odon Device was the only paper to provide detailed reasoning for device failure [27]. For all studies, the mode of delivery following device failure was reported which included forceps (*n=*21), ventouse (*n=*10), emergency Caesarean section (*n=*20), spontaneous vaginal birth (*n=*14) and non-investigational ventouse (*n=*10).

Only two studies mentioned ‘harm’, one stating ‘*device may have caused harm to the cervix*’ [28] in reference to their higher than expected cervical tear rate and the other reported ‘*no significant harm noted’* [37]. Only two studies documented anything specific regarding adverse events and/or safety reporting, both were the papers on the Odon Device. The pilot study paper stated that *‘No adverse outcomes were recorded in the six-week or one-year follow-up visits’,* that *‘No unexpected adverse events were reported.’* and *‘No long-term maternal or neonatal adverse outcomes were observed.’* [28]. The feasibility study paper went into significant detail outlining the neonatal and maternal Serious Adverse Events that arose during the trial [27].

### Assessment of study quality

The majority of studies were judged to be ‘unclear’ across all domains of bias except ‘random sequence generation and allocation concealment’ which had equal ‘unclear’ and ‘low-risk’ judgements (Table S2).

## Discussion

### Main findings

This systematic review summarised reporting standards of AVB in RCTs. Although the majority of the interventions or comparators were named, only half of the studies provided any description of how the interventions should be delivered and none were considered to be ‘precise’. The majority of papers did not discuss intervention standardization, focusing instead on criteria for device use (such as gestation or presentation of baby) rather than how the device should be used. Information regarding adherence was similarly poorly reported. Two-thirds of papers provided data regarding clinician expertise; however, this often was not detailed or specific. The overall lack of detail in the studies makes it hard to know exactly how procedures were intended to be performed and actually delivered, creating difficulties in interpreting results or replicating procedures in routine clinical practice.

Although this is the first study exploring reporting standards in RCTs of interventions for AVB, there have been several similar systematic reviews in RCTs of other invasive procedures (e.g. surgery) [[38], [39], [40], [41]]. Predominantly, these concluded that reporting standards were poor and surmised that this could be related to both a lack of uptake of CONSORT-NPT and a degree of ambiguity in the language used to define descriptions, standardization and adhernece [[38], [39], [40]]. It was particularly noted that despite provision of some intervention descriptions, they were often ambiguous and descriptions of all operative components were rarely included [38]. Moreover, it may not always be practical or desirable to provide ‘precise’ details of intervention delivery. In a multicentre pragmatic trial, for example, it may be very difficult to achieve standardization of every procedural component and step, and this is unlikely to reflect the heterogeneity of routine clinical practice. In reality, there may need to be a reasonable compromise between the level of detail and the flexibility permitted during procedural components, as complex interventions are influenced by patient, operator, device and centre-specific factors that may alter the way in which interventions can be delivered.

### Strengths and limitations

This review is the first published report detailing reporting standards relating to intervention description, standardization, adherence and expertise in AVB trials. The search strategy was wide and RCTs with a non-device comparator arm were also included. The review therefore provides a detailed inspection of reporting standards to provide an informative narrative to explain the reporting quality. Despite this, however, there are limitations. We only retrieved protocols or associated documents from authors specifically mentioning or referencing these within the trial report, so some may have been missed. Additionally, the majority of papers lacked methodological robustness, making it hard to relate positive findings to the wider research community. Finally, only nine papers were published after the release of the CONSORT-NPT guidelines in 2008 [42]. This is a key limitation as we were comparing papers to standards that were not available to the majority of authors at the time of publication. The present review however does highlight that the poor quality of reporting standards is constant, with no noticeable overall change in reporting quality since the introduction of these guidelines. The reasons for the lack of guideline adoption is likely to be multifactorial, relating to unclear, non-specific definitions and instructions, a lack of awareness amongst clinicians and trialists, and the lack of specificity of these guidelines towards interventions involving the use of devices.

### Interpretation

This review has highlighted the need for greater effort in the way devices are described, standardised and monitored in trial reports for AVB studies. This is likely to be the case for any device RCT, across all specialties. Trial protocols and reports should have greater consideration of the required details so that device use standards are clear enabling replication in routine practice.

### Conclusion

This paper presents a methodological review of reporting standards relating to intervention description, standardization, adherence and expertise for RCTs in AVB, comparing them with CONSORT-NPT guidance. Reporting standards in AVB trials are currently vague, lack transparency, detail and clarity, especially surrounding adherence. Although tools such as the CONSORT-NPT guidance attempt to address this issue, consideration of the details required specifically for interventions involving the use of devices is required. Such tools need to provide unambiguous and accessible detail by providing definitions of the key terms, such as standardization and adherence. Bespoke guidance for RCTs involving devices may help address this problem by describing and defining standards for trialists and clinicians, to improve methodological standards, facilitate replication of successful interventions in clinical practice, and reduce research waste.

## Contribution to authorship

EJH and NSB initiated and designed the study with methodological input from EL, JW, TJD and JFC. EJH, SR and NSB undertook data collection. EJH and NSB drafted the manuscript. All authors approved the final version of the manuscript.

## Details of ethics approval

Not applicable.

## Funding statement

This study was supported by the Bill & Melinda Gates Foundation [grant number OPP1184825 / INV-010180]. The views expressed are those of the author(s) and not necessarily those of the NIHR or the Department of Health and Social Care, and the MRC ConDuCT-II (Collaboration and innovation for Difficult and Complex randomised controlled Trials In Invasive procedures) Hub for Trials Methodology Research (MR/K025643/1).

## Declaration of Competing Interest

EJH, SR, JFC, TJD are employees of North Bristol NHS Trust, which receives funding from PROMPT Maternity Foundation (PMF) to pay part of their salaries. PMF has received funds from Becton Dickinson, manufacturer of the Odon Device to run pre-clinical simulation studies on the device. EL is an employee of the University of Bristol, which receives funding from PMF to pay part of EL’s salary. NSB is an MRC Clinician Scientist.
