# Supplementary material for: Exploring the reporting standards of RCTs involving invasive procedures for assisted vaginal birth: A systematic review
Source: Eur J Obstet Gynecol Reprod Biol. 2021 Jul;262:166–73. doi: 10.1016/j.ejogrb.2021.05.026 (PMC8250286; doi:10.1016/j.ejogrb.2021.05.026)
Supplement: Supplementary file 1 [file mmc1.docx]

**Table S1:**

**Medline Search Strategy**

|  | **Search terms** | **Number** |
| --- | --- | --- |
| 1 | exp "DELIVERY, OBSTETRIC"/ | 73069 |
| 2 | exp "LABOR, OBSTETRIC"/ | 44269 |
| 3 | PARTURITION/ | 7657 |
| 4 | (labor OR labour OR birth OR childbirth OR delivery).ti,ab | 659544 |
| 5 | (1 OR 2 OR 3 OR 4) | 706790 |
| 6 | exp "EXTRACTION, OBSTETRICAL"/ | 3266 |
| 7 | "OBSTETRICAL FORCEPS"/ | 1602 |
| 8 | (forceps).ti,ab | 9932 |
| 9 | (ventouse).ti,ab | 274 |
| 10 | ("suction cup").ti,ab | 278 |
| 11 | (kiwi OR malmstrom).ti,ab | 721 |
| 12 | (vacuum).ti,ab | 31586 |
| 13 | (odon).ti,ab | 43 |
| 14 | ((operative OR instrumental OR assisted) OADJ1 (delivery OR birth)).ti,ab | 3751 |
| 15 | (6 OR 7 OR 8 OR 9 OR 10 OR 11 OR 12 OR 13 OR 14) | 46256 |
| 16 | (randomized controlled trial).pt | 461986 |
| 17 | (controlled clinical trial).pt | 313627 |
| 18 | (multicenter study).pt | 234463 |
| 19 | (pragmatic clinical trial).pt | 784 |
| 20 | (randomis* OR randomiz* OR randomly).ti,ab | 771784 |
| 21 | (trial OR multicenter OR "multi center" OR multicentre OR "multi centre").ti | 216177 |
| 22 | NON-RANDOMIZED CONTROLLED TRIALS AS TOPIC/ | 355 |
| 23 | "FEASIBILITY STUDIES"/ | 58213 |
| 24 | "PILOT PROJECTS"/ | 106634 |
| 25 | (pilot OR feasibility).ti,ab | 264969 |
| 26 | (simulat*).ti,ab | 441283 |
| 27 | exp "SIMULATION TRAINING"/ | 6279 |
| 28 | (16 OR 17 OR 18 OR 19 OR 20 OR 21 OR 22 OR 23 OR 24 OR 25 OR 26 OR 27) | 1865422 |
| 29 | (5 AND 15 AND 28) | 1254 |
