# Supplementary material for: Exploring the reporting standards of RCTs involving invasive procedures for assisted vaginal birth: A systematic review
Source: Eur J Obstet Gynecol Reprod Biol. 2021 Jul;262:166–73. doi: 10.1016/j.ejogrb.2021.05.026 (PMC8250286; doi:10.1016/j.ejogrb.2021.05.026)
Supplement: Supplementary file 2 [file mmc2.docx]

**Table S2:**

**The Cochrane Collaboration’s Tool for Assessing Risk of Bias for Randomised Controlled Trials (*n*=35)**

|  | **Random sequence generation** | **Allocation concealment** | **Selective reporting** | **Blinding of participantand personnel** | **Blinding of outcome assessment** | **Incomplete outcome data** | **Other bias** |
| --- | --- | --- | --- | --- | --- | --- | --- |
| Afifi 1995 | L | L | U | H | H | L | U |
| Attilakos 2005 | H | H | H | H | H | L | H |
| Bofill 1996 | L | L | L | L | L | H | L |
| Carmody 1986 | L | L | L | L | L | L | L |
| Carmona 1995 | H | H | H | U | U | H | U |
| Chenoy 1992 | H | H | U | L | U | L | H |
| Cohn 1989 | L | L | L | L | L | L | H |
| Dell 1985 | L | L | U | U | U | L | U |
| Equy 2015 | L | L | U | H | U | U | H |
| Fitzpatrick 2003 | L | L | U | U | U | L | H |
| Gabrawi 1997 | L | L | U | U | U | H | U |
| Groom 2006 | L | L | U | U | U | H | U |
| Hammarstrom 1986 | H | H | U | U | U | H | H |
| Hebertson 1985 | H | H | U | U | U | L | U |
| Ismail 2008 | U | U | U | U | U | L | U |
| Johanson 1989 | L | L | L | H | H | L | U |
| Johanson 1993 | L | L | L | H | H | U | H |
| Khalid 2013 | U | U | U | U | H | U | H |
| Kuit 1993 | L | L | U | U | U | L | L |
| Lasbrey 1964 | U | U | U | H | H | U | U |
| Lee 1996 | U | U | U | U | U | L | L |
| Lim 1997 | U | U | U | L | U | U | U |
| Loghis 1992 | H | H | U | U | L | U | U |
| Maltau 1984 | U | U | L | U | L | U | H |
| Mola 2010 | L | L | U | L | U | U | U |
| Mustafa 2002 | H | H | H | U | U | U | H |
| Pliego Perez 2000 | U | U | U | U | U | U | U |
| Roshan 2005 | U | U | U | L | L | U | U |
| Shashank 2012 | U | U | U | U | U | H | U |
| Srisomboon 1998 | U | U | U | U | U | U | H |
| Suwannachat 2011 | L | L | U | U | U | L | L |
| Thiery 1987 | U | U | U | U | U | L | H |
| Vacca 1983 | U | U | U | H | U | U | U |
| Warwick 1993 | U | U | U | U | U | U | U |
| Weerasekera 2002 | U | U | U | U | U | H | U |
| Williams 1991 | U | U | U | U | U | H | U |
| Yancey 1991 | L | L | U | U | U | U | H |

**Evaluating bias for pilot and/or feasibility studies: low (L), high (H) and unclear (U) (*n*=1)**

|  | **Trial recruitment clearly defined** | **Protocol non-adherence clearly documented** | **Is there a primary outcome?** | **Are there clear objectives?** | **Are there clear progression or stop/go criteria** | **Other bias** |
| --- | --- | --- | --- | --- | --- | --- |
| Hotton 2020 | L | L | L | L | U | U |
| Schvartzman 2018 | L | U | U | L | U | U |
